# An Analysis of Intraoperative, Early Postoperative, and Late Postoperative Application of Negative Pressure Wound Therapy in Below-Knee Amputations in a Tertiary Care Center

**DOI:** 10.7759/cureus.93188

**Published:** 2025-09-25

**Authors:** Samir Deolekar, Ayush Mahajan, Shuddhabrata Panja, Uday Kumar

**Affiliations:** 1 General Surgery, Seth Gordhandas Sunderdas (GS) Medical College and King Edward Memorial (KEM) Hospital, Mumbai, IND

**Keywords:** below-knee amputation, diabetic foot, intraoperative vac, negative pressure wound therapy, vacuum-assisted closure (vac), wound healing enhancement

## Abstract

Background: Negative pressure wound therapy (NPWT) has been shown to improve wound healing through enhanced granulation tissue formation and exudate control. However, the timing of NPWT initiation in below-knee amputations (BKA) may significantly affect clinical outcomes, especially in resource-limited settings.

Objective: This study aimed to evaluate the effect of intraoperative, early postoperative (within seven days), and late postoperative (after seven days) initiation of NPWT on wound healing parameters and length of hospital stay in patients undergoing BKA.

Methods: A retrospective observational study was conducted on 75 patients who underwent BKA for diabetic foot complications. Patients were categorized into three groups (n = 25 each) based on the timing of NPWT initiation. Primary outcomes assessed were hospital stay duration and wound bed characteristics post-NPWT removal-granulation tissue percentage, slough percentage, wound contracture, and surrounding skin inflammation.

Results: Intraoperative NPWT led to significantly better outcomes: higher granulation tissue (96.8%), lower slough (2%), and shorter hospital stay (mean 1.24 days) compared to early (84% granulation, 9.6% slough, and 2.2 days stay) and late (69.2% granulation, 17.6% slough, and 4.24 days stay) NPWT. Wound contracture and skin inflammation differences were not statistically significant.

Conclusion: Early application of NPWT, especially intraoperatively, accelerates wound healing and reduces hospitalization time in BKA patients. These findings suggest a cost-effective strategy for improving patient outcomes and optimizing healthcare resource utilization in developing countries.

## Introduction

Negative pressure wound therapy (NPWT), also known as vacuum-assisted wound closure, is a wound management system that applies continuous or intermittent sub-atmospheric pressure to the wound surface through a sealed dressing system. This technique facilitates wound healing by promoting granulation tissue formation, enhancing local blood flow, and reducing wound area more effectively than conventional dressing systems. NPWT has also been shown in randomized trials and meta-analyses to accelerate wound closure and reduce the risk of infection in diabetic foot ulcers [1-5].

NPWT represents a significant advancement due to its multifaceted mechanisms of action. These include macro-deformation (wound shrinkage), micro-deformation at the foam-wound interface, removal of exudate and infectious material, and stimulation of angiogenesis, neurogenesis, and granulation tissue proliferation [6-8].

A standard NPWT system comprises an electronically controlled vacuum pump, a foam dressing, and a fenestrated drainage tube enclosed in an airtight seal with adhesive tape. The system typically applies suction in the range of 50 to 125 mmHg and is usually removed between the third and fifth day post-application [9].

Previous studies suggest that earlier initiation of NPWT-particularly within the first seven days of wound management-correlates with improved wound outcomes and shorter hospital stays compared to delayed initiation [10]. Although NPWT has been shown to improve wound healing in diabetic foot and surgical wounds, there is limited evidence on the impact of its timing following below-knee amputation.

This study aimed to compare the outcomes of intraoperative, early postoperative, and late NPWT initiation in patients undergoing below-knee amputation. The primary objective was to evaluate differences in hospital stay following NPWT removal. Secondary objectives included assessing wound granulation, slough, contracture, and periwound inflammation. We hypothesized that earlier NPWT initiation would be associated with improved wound healing and shorter hospitalization compared to later application.

## Materials and methods

Aim

The aim of the study is to assess the impact of NPWT timing (intraoperative, early postoperative, or late postoperative) on wound healing outcomes and hospital stay following below-knee amputation.

Primary objective

The primary objective is the length of hospital stay post-NPWT removal.

Secondary objective

The secondary objective is to evaluate the percentage of granulation tissue, percentage of slough, percentage of wound contracture, and presence of surrounding skin inflammation following NPWT removal.

Methodology

Study Design

This study is a single-center retrospective observational study in Seth Gordhandas Sunderdas (GS) Medical College and King Edward Memorial (KEM) Hospital, Mumbai (one year).

Study Groups

The study groups comprised 25 patients each in intraoperative, early postoperative (<7 days post op), and late postoperative (>7 days post op). No formal sample size calculation was performed, which we now acknowledge as a study limitation.

Inclusion criteria

The inclusion criteria include (1) age above 18 years, (2) patients with an open below-knee amputation stump (long posterior flap), and (3) patients who have undergone one session of NPWT lasting five days.

Exclusion criteria

The exclusion criteria include (1) cases with an acute thromboembolic phenomenon, (2) amputees who underwent stump closure as the study aimed to evaluate NPWT in open stumps, and (3) patients with incomplete records.

Study design

This was a single-center retrospective observational study, and its non-randomized nature should be considered when interpreting the findings. A polyurethane foam dressing with an occlusive drape was applied. Suction was set at 125 mmHg in continuous mode for a single five-day session, after which the wound was reassessed or earlier if the dressing became saturated with exudate. Granulation, slough, and contracture percentages were visually estimated by two independent surgeons using standardized photography and clinical notes with consensus agreement. Inter-observer consensus was achieved to minimize bias.

Contracture was defined as the percentage reduction in wound dimensions measured from standardized wound photographs taken before and after NPWT application. The study was approved by the Institutional Ethics Committee (Approval Number: EC/146/2022).

Statistical analysis

Normality was tested prior to analysis. One-way ANOVA was used for continuous variables; where assumptions were borderline, Kruskal-Wallis testing produced comparable results. Categorical outcomes were analyzed with chi-squared or Fisher’s exact test.

## Results

Gender distribution

Among patients undergoing below-knee amputation, men were 47 (62.66 %) in number and women were 28 (37.33%) in number. The intraoperative group had 60% men. The early postoperative group and the late postoperative group had 64% men each.

Age distribution

The study comprised 25 patients in each group, hence a total of 75 patients. The most common age group of patients undergoing below-knee amputation was between 39-48 (29.3%) and 49-58 (29.3%). The intraoperative group had the highest number of patients from the age group of 39-48 (40%), early post op had the age group of 39-48 (28%), and the late postoperative group had the age group of 49-58 (36%).

Length of hospital stay post-NPWT removal

**Figure 1:**
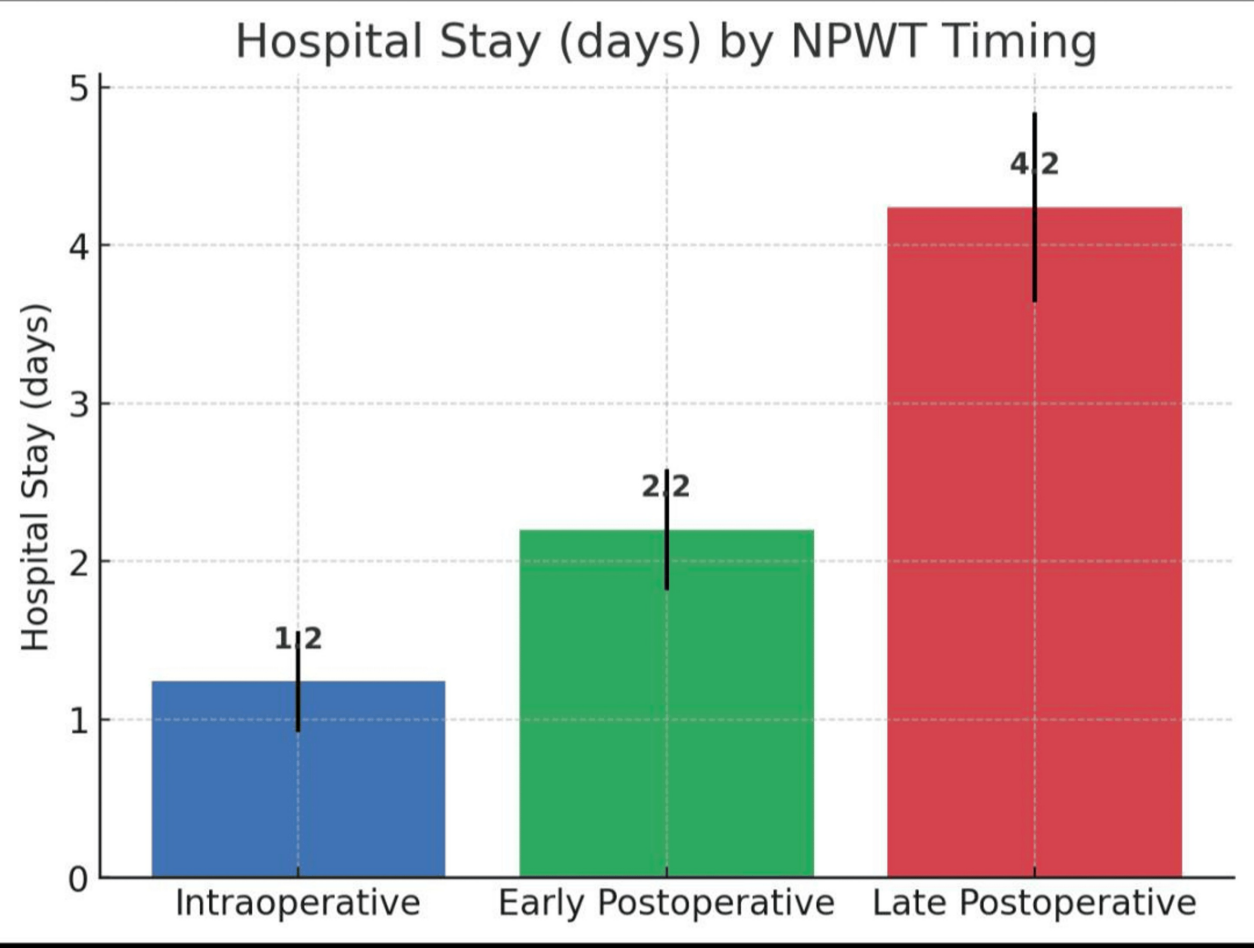
Length of stay after NPWT removal NPWT: negative pressure wound therapy

The intraoperative group had the shortest stay (1.2 days, 95% CI 0.9-1.6) compared with early postoperative (2.2 days, 95% CI 1.8-2.6) and late postoperative (4.2 days, 95% CI 3.6-4.8). The difference was highly significant (ANOVA F = 49.74, p = 2.7 × 10⁻¹⁴) and clinically meaningful, reflecting a reduction of nearly three days with earlier NPWT initiation (Table 1, Figure 1).

**Table 1:** Length of stay post VAC removal VAC: vacuum-assisted closure

Days post VAC	Early postoperative		Intraoperative		Late postoperative		p-value
	Mean	SD	Mean	SD	Mean	SD	
	2.2	0.91	1.2	0.78	4.2	1.45	2.7 × 10⁻¹⁴

Percentage of granulation tissue of NPWT removal

**Figure 2:**
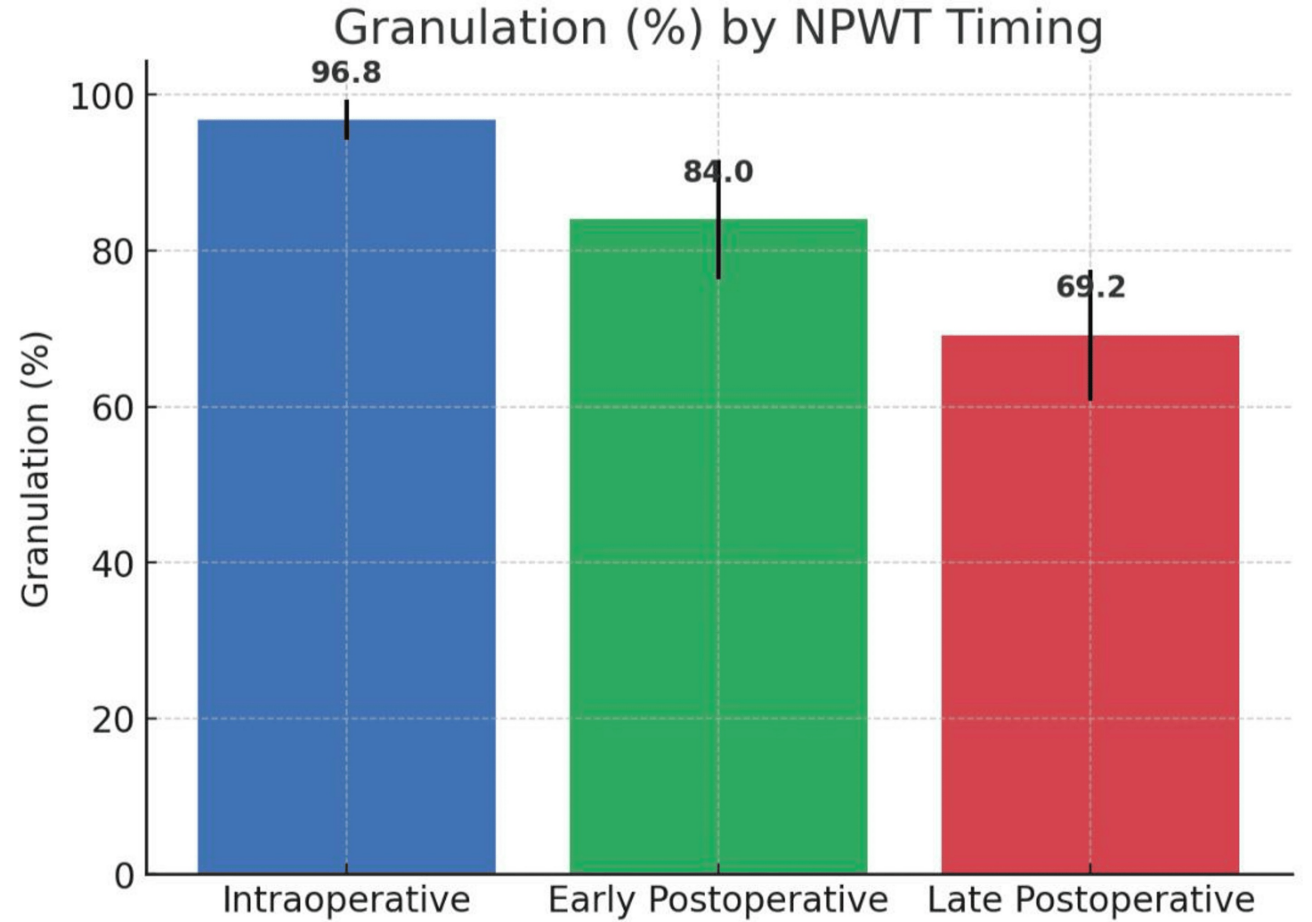
Percentage of granulation by NPWT timing NPWT: negative pressure wound therapy

Mean granulation was highest in the intraoperative group (96.8%, 95% CI 94.2-99.4) compared to the early postoperative (84.0%, 95% CI 76.3-91.7) and late postoperative (69.2%, 95% CI 60.8-77.6) groups. This difference was statistically significant (ANOVA F = 17.76, p = 5.4 × 10⁻⁷) and clinically relevant, as earlier NPWT initiation was associated with markedly better wound bed readiness (Table 2, Figure 2).

**Table 2:** Percentage of granulation tissue of NPWT removal NPWT: negative pressure wound therapy

% of granulation	Early postoperative		Intraoperative		Late postoperative		p-value
	Mean	SD	Mean	SD	Mean	SD	
	84.0	12.0	96.8	6.4	69.2	13.0	p = 5.4 × 10⁻⁷

Percentage of slough

**Figure 3:**
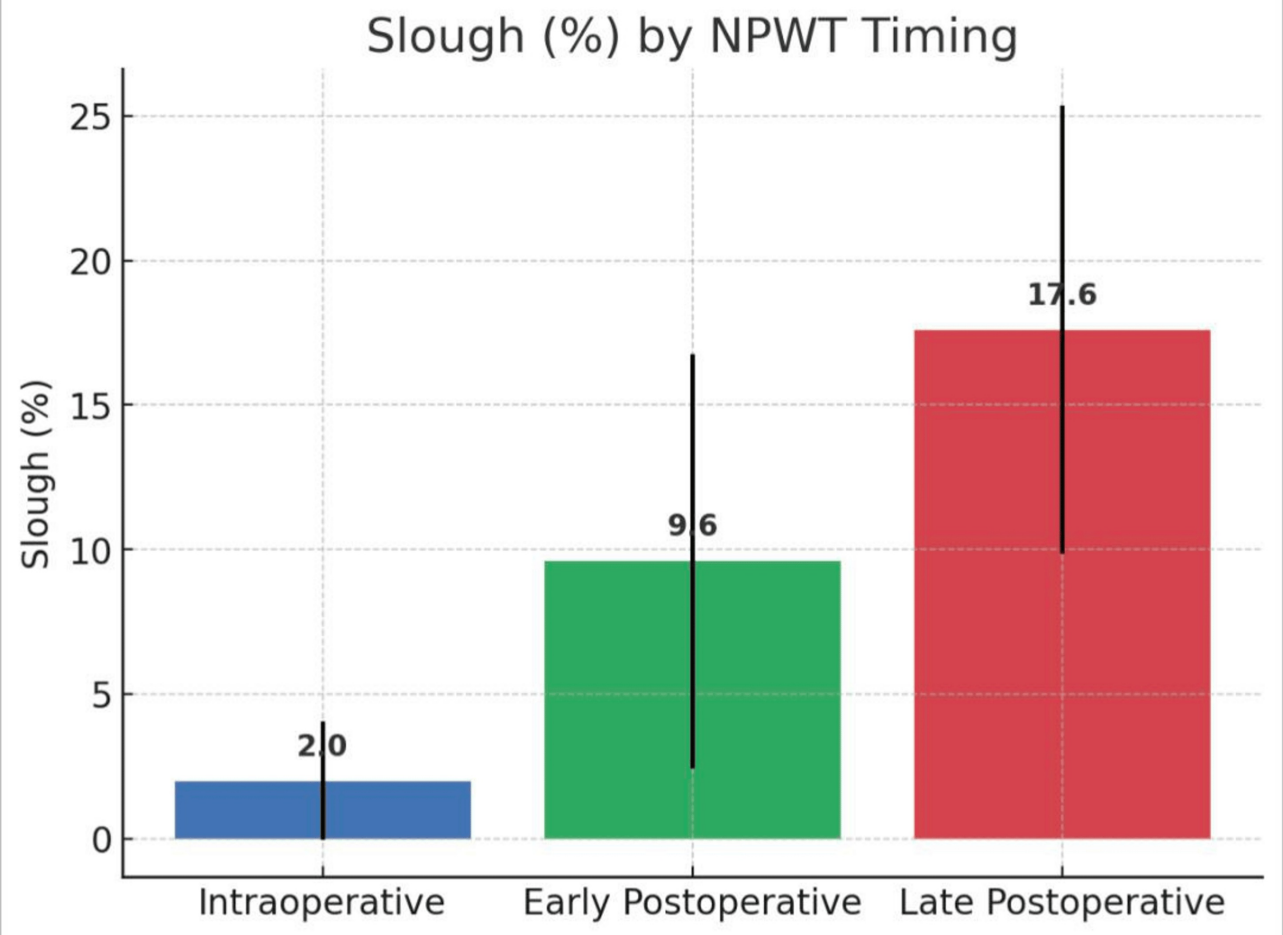
Percentage of slough NPWT: negative pressure wound therapy

Slough burden was lowest in the intraoperative group (2.0%, 95% CI -0.1-4.1) compared to early postoperative (9.6%, 95% CI 2.4-16.8) and late postoperative (17.6%, 95% CI 9.9-25.4). The difference was significant (ANOVA F = 6.72, p = 0.0021) (Table 3, Figure 3).

**Table 3:** Percentage of slough

% of slough	Early postoperative		Intraoperative		Late postoperative		p-value
	Mean	SD	Mean	SD	Mean	SD	
	9.6	12.0	2.0	4.8	17.6	17.6	0.0021

Percentage of wound contracture

**Figure 4:**
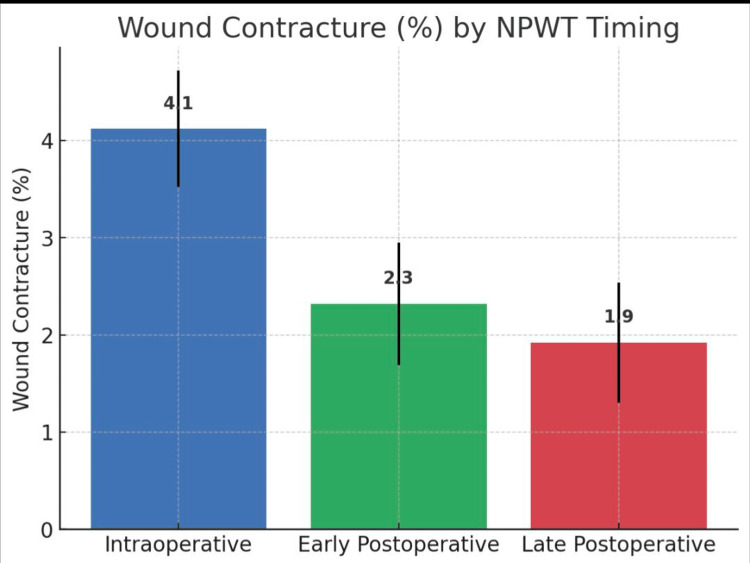
Wound contracture on the day of NPWT removal NPWT: negative pressure wound therapy

Contracture was modest across all groups, with means of 4.1% (95% CI 3.5-4.7) intraoperatively, 2.3% (95% CI 1.7-3.0) early postoperatively, and 1.9% (95% CI 1.3-2.5) late postoperatively. This difference reached statistical significance (ANOVA F = 15.46, p = 2.6 × 10⁻⁶), though the clinical relevance was less pronounced (Table 4, Figure 4).

**Table 4:** Percentage of wound contracture

% of contracture	Early postoperative		Intraoperative		Late postoperative		p-value
	Mean	SD	Mean	SD	Mean	SD	
	2.3	1.5	4.1	1.2	1.9	1.9	p = 2.6 × 10⁻⁶

Surrounding skin inflammation

**Figure 5:**
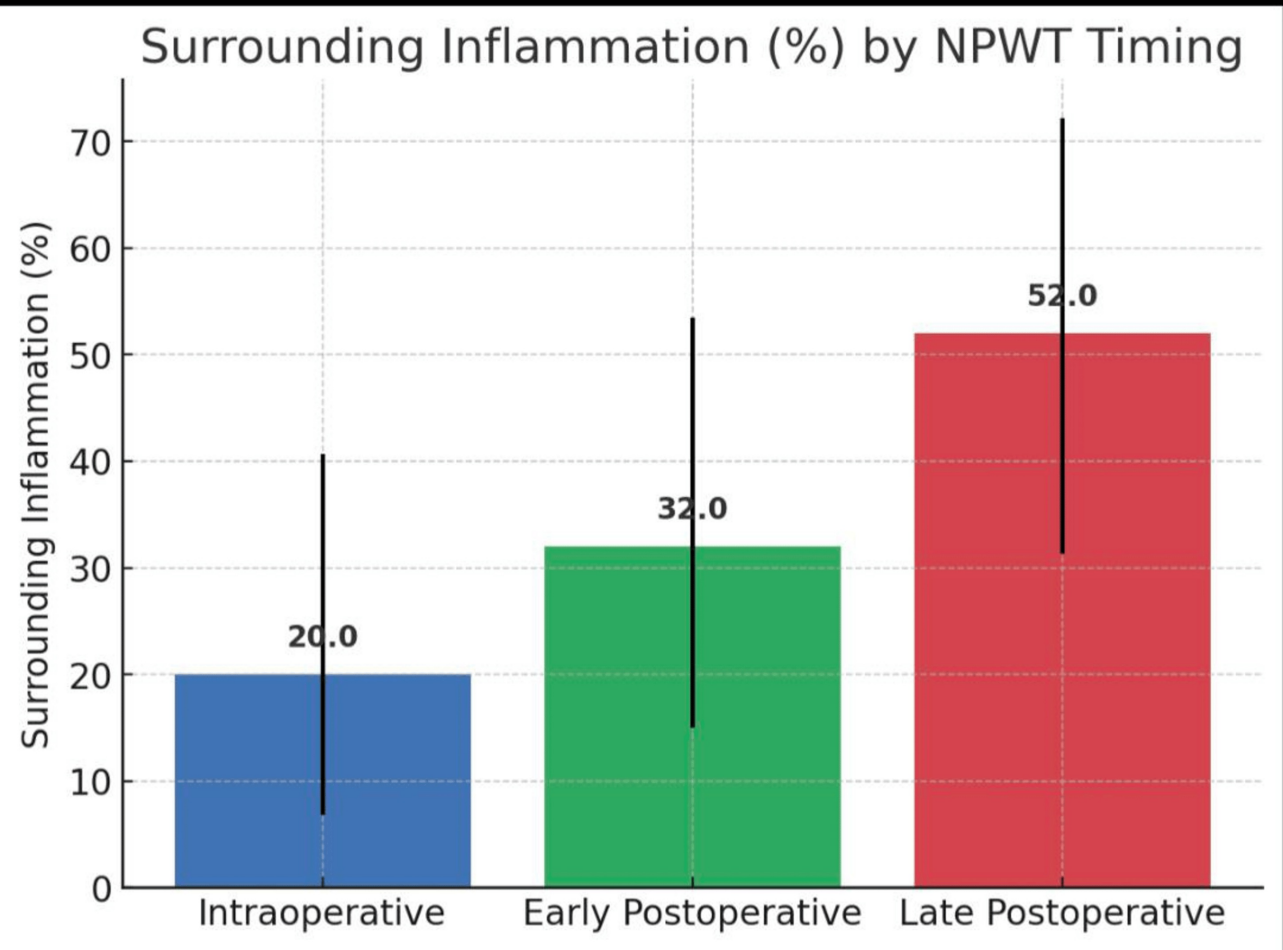
Surrounding skin inflammation NPWT: negative pressure wound therapy

Inflammation rates were 20.0% (95% CI 6.8-40.7) in the intraoperative group, 32.0% (95% CI 15.0-53.5) in the early postoperative group, and 52.0% (95% CI 31.3-72.2) in the late postoperative group. The association approached but did not reach significance (χ² = 5.77, df = 2, p = 0.0559; Cramér’s V = 0.277) (Table 5, Figure 5).

**Table 5:** Surrounding skin inflammation

Surrounding inflammation	Early postoperative		Intraoperative		Late postoperative		p-value
	Number	%	Number	%	Number	%	
Yes	8	32.0	5	20.0	13	52.0	0.0559
No	17	68.0	20	80.0	12	48.0	

## Discussion

NPWT has been widely studied for its ability to optimize wound healing by promoting granulation tissue formation, reducing exudate, and stabilizing the wound environment. In this study, NPWT applied intraoperatively or within the first postoperative week was associated with significantly higher granulation percentages, lower slough burden, and shorter post-NPWT hospital stays compared to later initiation [11,12].

The finding that intraoperative NPWT was associated with the highest granulation (96.8%) and lowest slough (2.0%) supports prior evidence that earlier initiation promotes faster wound bed preparation [13]. Similarly, Liu et al. [14] and Cho et al. [15] demonstrated in systematic reviews that NPWT accelerates wound closure and decreases infection risk in diabetic foot wounds. Our results extend these observations by showing that the timing of NPWT application after below-knee amputation may influence wound bed readiness and hospital resource utilization [13-15].

Hospital stay after NPWT removal was shorter in the intraoperative and early groups compared with late NPWT initiation. This finding is clinically relevant, especially in resource-limited healthcare settings, where earlier discharge can reduce costs and improve bed turnover. While the association between earlier NPWT and reduced hospitalization is consistent with Kaplan et al. [9] and Baharestani et al. [16], it should be interpreted cautiously given the retrospective study design [16].

Unlike granulation and slough, wound contracture and surrounding inflammation did not differ significantly across groups. This suggests that NPWT’s benefits in this context may be more strongly related to granulation and wound bed cleanliness rather than contraction or periwound inflammation.

One potential drawback of NPWT is the risk of hypergranulation tissue, particularly with prolonged therapy or higher pressures. Although hypergranulation was not observed in our cohort, this remains a theoretical risk and warrants monitoring in clinical practice.

Limitations of the study

This was a single-center retrospective study with a limited sample size, and therefore, results should be considered preliminary. The retrospective nature, absence of sample size calculation, and lack of adjustment for confounders (glycemic control, vascular status, and infection severity) are acknowledged as limitations.

Small Sample Size (n = 75)

The small sample size is underpowered for some outcomes, such as surrounding inflammation.

Wound Assessment Methods

Granulation, slough, and contracture percentages were estimated visually from wound photographs and records, introducing subjectivity despite inter-observer consensus.

No Long-Term Follow-Up

We could not assess stump healing beyond the immediate NPWT episode or evaluate re-amputation/secondary closure outcomes.

No Sample Size Calculation Performed A Priori

This has been acknowledged as a methodological limitation.

## Conclusions

The results of this study help us come to the conclusion that early NPWT, particularly intraoperative application, is associated with improved granulation, reduced slough, and shorter hospital stays. However, these findings must be interpreted with caution, given the retrospective design and small sample size. Larger, prospective multicenter trials are necessary to confirm these observations.
